# Effects of *Salvia sclarea* on chronic immobilization stress induced endothelial dysfunction in rats

**DOI:** 10.1186/1472-6882-14-396

**Published:** 2014-10-14

**Authors:** Hyo Jung Yang, Ka Young Kim, Purum Kang, Hui Su Lee, Geun Hee Seol

**Affiliations:** Department of Basic Nursing Science, School of Nursing, Korea University, 145 Anam-ro, 136-701 Seongbuk-gu, Seoul, Republic of Korea

**Keywords:** *Salvia sclarea*, Chronic immobilization stress, Endothelial dysfunction, Oxidative stress

## Abstract

**Background:**

Although *Salvia sclarea* (clary sage) is widely used in aromatherapy and has antioxidant and antimicrobial properties, its mechanisms of action remain poorly understood. We therefore assessed whether clary sage is effective in treating endothelial dysfunction induced by chronic immobilization stress in rats.

**Methods:**

Rats were intraperitoneally injected with almond oil, clary sage oil (5%, 10% or 20%), or nifedipine once daily, followed by immobilization stress (2 h/day) for 14 days. Systolic blood pressure (SBP) and heart rate (HR) were measured, as were serum concentrations of corticosterone (CORT); a biomarker of chronic stress, malondialdehyde (MDA); a biomarker of oxidative stress. Nitric oxide production was assessed by nitrite assays, and eNOS level, a biomarker of endothelial dysfunction, was measured by western blotting. Endothelial dysfunction was also assayed by measuring the effect of clary sage on the contraction of rat aortae.

**Results:**

Treatment with 5% (*p* = 0.029), 10% (*p* = 0.008), and 20% (*p* = 0.008) clary sage significantly reduced SBP and treatment with 20% clary sage significantly reduced HR (*p* = 0.039) compared with the chronic immobilization stress group. Clary sage decreased CORT serum concentration (10%, *p* = 0.026; 20%, *p* = 0.012) and MDA (10%, *p* = 0.007; 20%, *p* = 0.027), findings similar to those observed with nifedipine. In addition, 20% clary sage significantly increased nitric oxide production (*p* <0.001) and eNOS expression level (*p* <0.001) and relaxed aortic rings in rats subjected to chronic immobilization stress.

**Conclusions:**

Clary sage treatment of rats subjected to immobilization stress contributed to their recovery from endothelial dysfunction by increasing NO production and eNOS level as well as by decreasing oxidative stress. Appropriate concentration of clary sage may result in recovery from endothelial dysfunction. These findings indicate that clary sage oil may be effective in the prevention and treatment of stress-induced cardiovascular diseases.

## Background

Endothelial dysfunction is characterized by reduced vasodilation, both generally and in response to specific stimuli, as well as a proinflammatory and prothrombic state [1]. Endothelial dysfunction has been implicated in the pathogenesis of several cardiovascular diseases, including hypertension, atherosclerosis, coronary artery disease, and chronic heart failure. Chronic immobilization and vascular oxidative stress lead to endothelial dysfunction and systolic hypertension, activating the angiotensin II and AT1 receptor signaling pathway [2]. Responses to chronic stress include increased production of corticotropin-releasing hormone and increased central sympathetic activity, inducing cardiovascular reactivity including blood pressure and heart rate [3]. Furthermore, chronic immobilization stress induces working memory impairment, anxiety and depressive-like behavior [4]. Oxidative stress also causes endothelial dysfunction by impairing endothelium-dependent relaxation and by inducing intracellular calcium overload and DNA fragmentation in hypertensive models [5].

Endothelial dysfunction in hypertensive patients has also been associated with decreased production of nitric oxide (NO), a key vasodilator released by the endothelium. NO has been associated with various endothelial functions, including the regulation of vascular tone, platelet aggregation, and vascular smooth muscle cell proliferation [6]. Therefore, endothelial dysfunction may result from the reduced activity of endothelial NO synthase and the resulting decreased bioavailability of NO [1].

*Salvia sclarea* (Clary sage) is an important aromatic plant of the mint family that has various pharmacological properties, including antioxidant and antimicrobial activities [7]. Inhalation of clary sage has been shown to induce relaxation in patients with urinary incontinence by reducing systolic blood pressure [8]. Blended essential oils containing clary sage, lavender and marjoram have been found to relieve pain in women with dysmenorrhea [9]. In rats, clary sage has anti-depressant activity through modulation of the dopaminergic pathway [10]. Despite its widespread use in aromatherapy and its therapeutic activities, little is known about the mechanisms of action of clary sage. We therefore assessed whether clary sage is effective in the treatment of rats with endothelial dysfunction induced by chronic immobilization stress.

## Methods

### Rat model of immobilization stress

Male Sprague–Dawley rats, aged 8 weeks and weighing 200–250 g (Samtaco INC., Osan, Korea), were acclimatized to standard laboratory conditions for 3–5 days. All experimental procedures were conducted in accordance with guidelines relevant to the care of experimental animals, as approved by the Animal Research Committee of Korea University (approval no. KUIACUC-2012-181), informed by the Guide for the Care and Use of Laboratory Animals published by the US National Institutes of Health (NIH Publication No. 85–23; revised 1996). Rats were randomized into five groups of seven to nine each and intraperitoneally injected with 0.1 ml/100 g body weight almond oil, clary sage oil (5%, 10%, or 20%, vol/vol), or nifedipine (10 mg/kg) once daily prior to the induction of immobilization stress by restraints. Rats were immobilized for 2 hours per day for 14 days using an adjustable restraining cage [2].

### Materials

Clary sage oil was supplied by Aromarant Co. Ltd. (Rottingen, Germany) and diluted in almond oil. Nifedipine was purchased from Sigma-Aldrich (Steinheim, Germany), and dissolved in dimethyl sulfoxide.

### Measurement of systolic blood pressure (SBP) and heart rate (HR)

The SBP and pulse signal level (heart rate) were measured one day before the first stress day and one day after the last stress day using a tail cuff and pulse transducer (ADInstruments, Sydney, Australia). Five measurements were taken at each time point and the mean systolic blood pressure calculated.

### Blood sampling and serum corticosterone (CORT) assay

Blood samples were obtained the day after completion of stress treatment by cardiac puncture from rats anesthetized with isoflurane (Hana Pharm. Co., Ltd., Seoul, Korea). Samples were centrifuged for 15 min at 13,000 × g within 30 min of collection and stored at −80°C. CORT was assayed in serum using an ELISA kit (Assay Designs Inc. Ann Arbor, MI) according to the manufacturer’s instructions.

### Malondialdehyde (MDA) and nitrite assays

Serum MDA concentration was measured spectrophotometrically at 532 nm as thiobarbituric acid reactive substances using a colorimetric/fluorometric assay kit according to the manufacturer’s instructions (Biovision, CA, USA). Serum nitrite concentration was measured by the Griess reaction. Briefly, 100 μl of Griess reagent (1% sulfanilamide, 0.1% naphthylethylenediamide in 5% phosphoric acid) was mixed with 50 μl of serum and incubated for 10 minutes at room temperature, and the optical density at 540 nm was determined using a microplate reader (BMG Labtech, Ortenberg, Germany).

### Western blot analysis

Rat aorta homogenates were separated on 10% sodium dodecyl sulfate (SDS)-polyacrylamide gels. The proteins were electrophoretically transferred onto nitrocellulose membranes and blocked for 30 min at room temperature. Membranes were incubated overnight at 4°C with primary antibodies to eNOS and glyceraldehyde-3-phophate dehydrogenase (GAPDH) and then with horseradish peroxidase-conjugated secondary antibody for 1 h at room temperature. The signal was visualized by enhanced chemiluminescence (ECL) reagents according to the manufacturer’s protocol.

### Preparation of isolated rat aortic rings

Isolated rat thoracic aortae were dissected to remove connective and fatty tissue and cut into rings about 4 to 5 mm in length. Rings were mounted in organ baths containing Krebs- Henseleit solution (118.3 mM NaCl, 4.78 mM KCl, 25 mM NaHCO_3_, 1.22 mM KH_2_PO_4_, 11.1 mM glucose, 2.5 mM CaCl_2_, and 1.2 mM MgCl_2_). The baths were maintained at 37°C and aerated with 95% O_2_ and 5% CO_2_ throughout the experiment. Resting tension (0.7-0.9 g) was applied for 1 h.

### Statistical analysis

Data were expressed as mean ± standard error of the mean (SEM). Differences among treatment groups were evaluated by one-way ANOVA, followed by LSD post hoc test. A *P* value <0.05 was considered statistically significant.

## Results

### Effects of clary sage on SBP and HR in chronic immobilization stress induced rat

**Figure 1:**
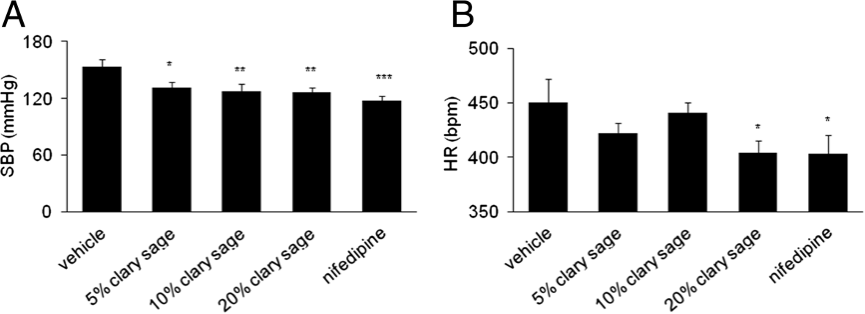
**Effects of clary sage on SBP and HR in chronic immobilization stress induced rats.** Rats were exposed to immobilization stress (2 h/day) for 14 days, with administration of almond oil, clary sage (5%, 10%, or 20%) or nifedipine (10 mg/kg) once daily. **(A)** SBP and **(B)** HR were measured using a tail cuff and pulse transducer. Data represent mean values ± SEM (n = 7-9 per group). *P <0.05, **P <0.01, ***P <0.001 compared to the stress vehicle group.

As expected, SBP was significantly higher in rats immobilized 2 hours per day for 14 days compared with control rats (*P* <0.001) (data not shown). SBP was significantly decreased by treatment with 5% (132.02 ± 16.59 mmHg, *P* = 0.029), 10% (127.97 ± 22.70 mmHg, *P* = 0.008) and 20% (127.09 ± 12.18 mmHg, *P* = 0.008) clary sage compared with the stress group (152.82 ± 23.08 mmHg) after completion of 2-week immobilization (Figure 1A). Furthermore, 20% clary sage effectively reduced HR (404.54 ± 28.22 bpm, *P* = 0.039) compared with the stress group (450.85 ± 61.04 bpm) (Figure 1B). Nifedipine significantly decreased SBP (117.60 ± 15.71 mmHg, *P* <0.001) and HR (403.01 ± 50.61 mmHg, *P* = 0.028).

### Effect of clary sage on CORT in chronic immobilization stress induced rats

**Figure 2:**
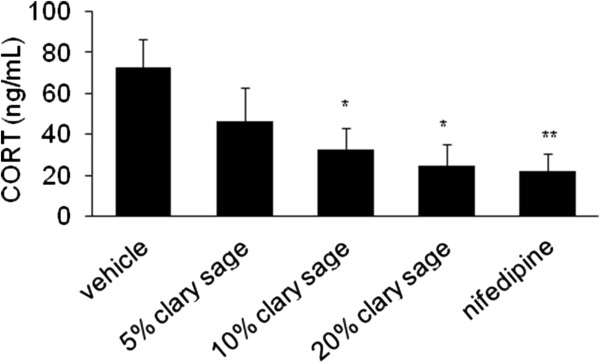
**Effect of clary sage on CORT in chronic immobilization stress induced rats.** Serum CORT concentrations were assayed by ELISA. Data represent mean values ± SEM (n =7-9 per group). *P <0.05, **P <0.01 compared to the stress vehicle group.

Compared with chronic immobilization stress induced rats (72.82 ± 36.81 ng/mL), treatment with 10% (32.27 ± 28.45 ng/mL, *P* = 0.026) and 20% (24.83 ± 28.27 ng/mL, *P* = 0.012) clary sage significantly reduced serum CORT concentration. Similarly, nifedipine significantly reduced serum CORT concentration (21.86 ± 23.15 ng/mL, *P* = 0.008) (Figure 2).

### Effect of clary sage on MDA in chronic immobilization stress induced rats

**Figure 3:**
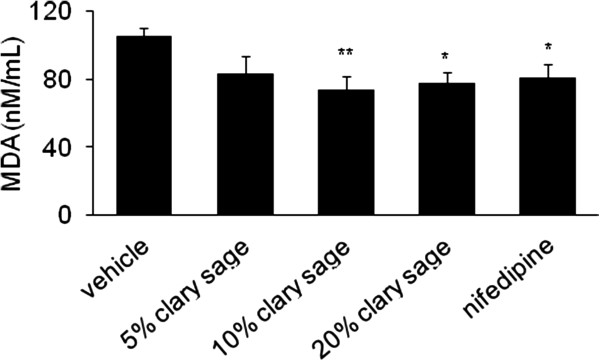
**Effect of clary sage on MDA in chronic immobilization stress induced rats.** MDA was assessed in serum as a biomarker for oxidative stress. Data represent mean values ± SEM (n = 7-9 per group). *P <0.05, **P <0.01 compared to the stress vehicle group.

To investigate whether clary sage was associated with oxidative stress in chronic immobilization, we measured serum concentrations of MDA, a biomarker of oxidative stress. Clary sage decreased the serum level of MDA compared with immobilization stress group (105.27 ± 12.45 nM/mL) and this reduction was significant in 10% (73.89 ± 21.07 nM/mL, *P* = 0.007) and 20% (78.00 ± 13.51 nM/mL, *P* = 0.027) clary sage groups. Nifedipine showed a significant decrease compared with chronic immobilization stress group (80.87 ± 19.56 nM/mL, *P* = 0.037) (Figure 3).

### Effect of clary sage on nitrite, eNOS and contraction of aortic rings in chronic immobilization stress induced rats

**Figure 4:**
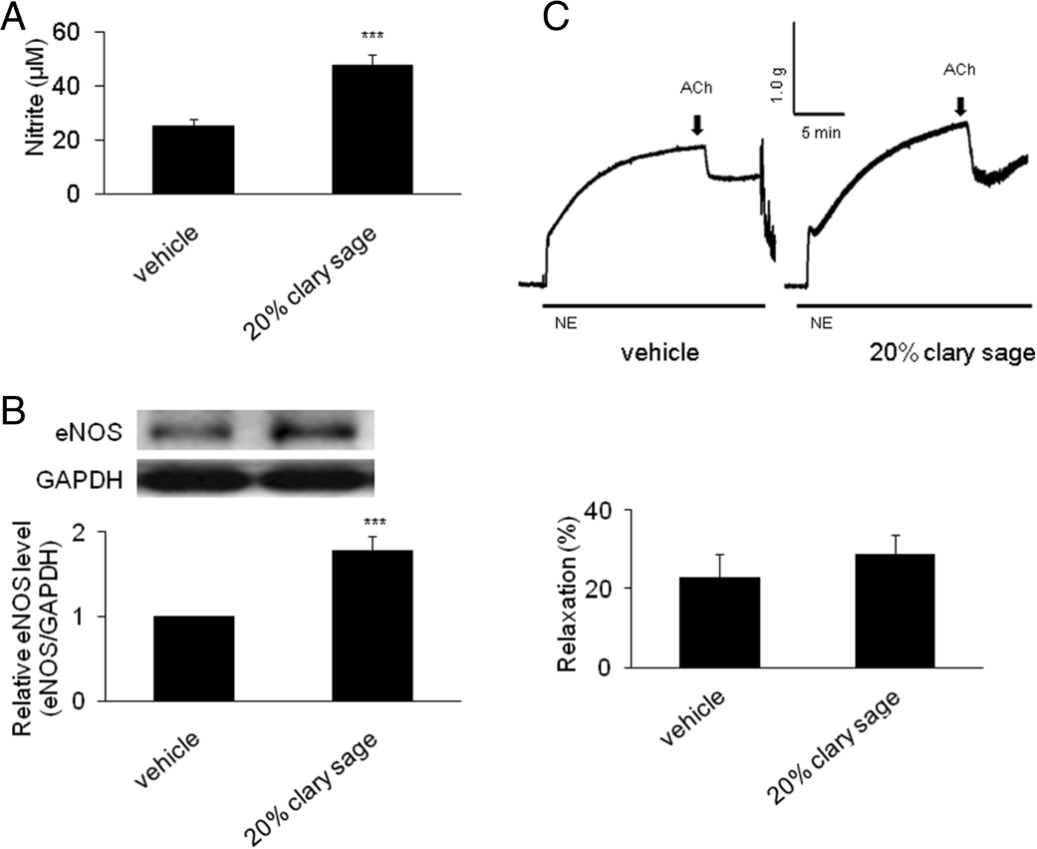
**Effect of clary sage on nitrite, eNOS and contraction of aortic rings in chronic immobilization stress induced rats. (A)** Serum nitrite level was measured as a marker of endothelial dysfunction. **(B)** eNOS level was analyzed by western blotting, with GAPDH used as an internal control. **(C)** Vasorelaxation in response to 10 μM acetylcholine was assessed in aortic rings of rats treated with clary sage or vehicle following contractions induced by 10 μM norepinephrine. Data represent mean ± SEM (n = 7-9 per group). ***P <0.001 compared with the stress vehicle group.

To demonstrate endothelial dysfunction, we focused on 20% clary sage which was the most efficient concentration in this study. Serum nitrite concentration, a biomarker of endothelial dysfunction, was significantly higher in immobilized rats administered 20% clary sage (47.88 ± 15.97 *μ*M, *P* <0.001) than in stressed rats (25.76 ± 8.37 *μ*M) (Figure 4A). Furthermore, 20% clary sage significantly increased eNOS expression levels 1.79-fold (*P* <0.001) in rats subjected to chronic immobilization stress (Figure 4B). Aortic rings obtained from rats treated with clary sage showed a greater relaxation tendency in response to ACh than vehicle treated rats (Figure 4C).

## Discussion

Exposure to chronic stress contributes to the development of hypertension and immobilization stress has been found to increase plasma concentrations of adrenocorticotropic hormone, corticosterone, norepinephrine and epinephrine, resulting in increased blood pressure and heart rate [11, 12]. This study was designed to investigate the protective effects of clary sage against chronic immobilization stress. We found that clary sage significantly reduced SBP and HR in rats subjected to immobilization stress. Furthermore, clary sage significantly decreased plasma CORT concentrations. The activities of clary sage were similar to those of the calcium channel blocker nifedipine, which is used to treat high blood pressure [13] and has been found to improve endothelial function by reducing oxidative stress and suppressing endothelial progenitor cell apoptosis [14].

Oxidative stress is increased in chronic stress conditions, including psychological stress in humans and restraint stress in animals [15]. Excessive production of ROS has been reported to causes hypertension and scavenging of ROS has been shown to reduce blood pressure [16]. Chronic immobilization stress by restraint has been found to alter the prooxidant-antioxidant balance, leading to the development of various pathological states such as mitochondrial dysfunction, disruption of energy pathways, neuronal damage, impaired neurogenesis and induction of signaling events in apoptotic cell death [4, 15]. Interestingly, we found that 20% clary sage decreased oxidative stress and increased NO production and eNOS expression in chronic immobilization induced rats.

Enhanced ROS production and destruction of the antioxidant system have been associated with endothelial dysfunction in cardiovascular diseases [5]. Normal endothelium sustains vascular tone and structure by modulating the balance between vasodilators such as NO and prostacyclin, and vasoconstrictors such as endothelin-1 and angiotensin II [5]. Reduced NO bioavailability, due to decreased NO production and/or increased NO inactivation, has been shown to aggravate endothelial dysfunction. The serine/threonine kinase Akt increases NO production by endothelial cells by activating eNOS [17].Oxidative stress causing impaired NO bioavailability also contributes to endothelial dysfunction. Decreased NO and oxidative excess may induce matrix metalloproteinases (MMPs), usually MMP-2 and MMP-9, which break down the fibrous cap containing collagen, elastin, and proteoglycans, resulting in endothelial dysfunction [1]. Furthermore, plasma MDA and ADMA levels were effectively increased and plasma nitrate concentrations significantly reduced in patients with overt hypothyroidism, a mechanism closely implicated in endothelial dysfunction [6]. Our findings suggest that chronic immobilization stress may cause endothelial dysfunction and systolic hypertension through vascular oxidative stress. Excess generation of ROS and oxidative stress has been implicated in endothelial dysfunction through the inactivation of NO. In this study, clary sage may play an important role in treating endothelial dysfunction by regulating oxidative stress and NO.

## Conclusions

Clary sage treatment of rats subjected to immobilization stress contributed in recovery from endothelial dysfunction by decreasing oxidative stress and increasing NO production and eNOS expression. Appropriate concentrations of clary sage may result in recovery from endothelial dysfunction. These findings indicate that clary sage oil may be effective in the prevention and treatment of stress-induced cardiovascular diseases.

## Abbreviations

SBP: Systolic blood pressure
HR: Heart rate
NO: Nitric oxide
eNOS: Endothelial nitric oxide synthase
CORT: Corticosterone
MDA: Malondialdehyde
MMPs: Matrix metalloproteinases.
